# Intravoxel Incoherent Motion Imaging on Sacroiliitis in Patients With Axial Spondyloarthritis: Correlation With Perfusion Characteristics Based on Dynamic Contrast-Enhanced Magnetic Resonance Imaging

**DOI:** 10.3389/fmed.2021.798845

**Published:** 2022-01-26

**Authors:** Chang Guo, Kai Zheng, Qiang Ye, Zixiao Lu, Zhuoyao Xie, Xin Li, Yinghua Zhao

**Affiliations:** Department of Radiology, Academy of Orthopedics, The Third Affiliated Hospital, Southern Medical University, Guangzhou, China

**Keywords:** spondyloarthritis, magnetic resonance imaging, diffusion-weighted imaging, intravoxel incoherent motion, dynamic contrast-enhanced

## Abstract

**Background:**

To prospectively explore the relationship between intravoxel incoherent motion (IVIM) diffusion-weighted imaging (DWI) and dynamic contrast-enhanced MRI (DCE-MRI) parameters of sacroiliitis in patients with axial spondyloarthritis (axSpA).

**Methods:**

Patients with initially diagnosed axSpA prospectively underwent on 3.0 T MRI of sacroiliac joint (SIJ). The IVIM parameters (*D, f*, D^*^) were calculated using biexponential analysis. *K*^trans^, *K*_ep_, *V*_e_, and *V*_p_ from DCE-MRI were obtained in SIJ. The uni-variable and multi-variable linear regression analyses were used to evaluate the correlation between the parameters from these two imaging methods after controlling confounders, such as bone marrow edema (BME), age, agenda, scopes, and localization of lesions, and course of the disease. Then, their correlations were measured by calculating the Pearson's correlation coefficient (*r*).

**Results:**

The study eventually enrolled 234 patients (178 men, 56 women; mean age, 28.51 ± 9.50 years) with axSpA. With controlling confounders, *D* was independently related to *K*^trans^ (regression coefficient [*b*] = 27.593, *p* < 0.001), *K*_ep_ (*b* = −6.707, *p* = 0.021), and *V*_e_ (*b* = 131.074, *p* = 0.003), whereas *f* and D^*^ had no independent correlation with the parameters from DCE MRI. The correlations above were exhibited with Pearson's correlation coefficients (*r*) (*r* = 0.662, −0.408, and 0.396, respectively, all *p* < 0.001).

**Conclusion:**

There were independent correlations between *D* derived from IVIM DWI and *K*^trans^, *K*_ep_, and *V*_e_ derived from DCE-MRI. The factors which affect their correlations mainly included BME, gender, and scopes of lesions.

## Introduction

SpondyloArthritis (SpA) is the chronic inflammatory arthritis that mainly involves sacroiliac and facet joints before the onset of 40 years old. The disease alternates between the active and chronic phases. The chronic complaints, such as spinal rigidity, deformity, disability, and so on, seriously affect quality of life of the patient (1–3). Clinically, the main manifestations of axial SpA (axSpA) are inflammatory back pain and enthesitis, such as ankylosing spondylitis (AS). In addition, SpA involves peripheral joints and is classified as peripheral SpA (such as, reactive arthritis, psoriatic arthritis, and arthritis associated with inflammatory bowel disease) (4). Sacroiliitis is a hallmark of axSpA and is an important index for the determination of the activity stage in patients with SpA (5).

MRI has been used to provide a comprehensive assessment of this disease activity in patients with SpA (6). However, based on the conventional MR sequence, Spondyloarthritis Research Consortium of Canada (SPARCC) scoring system is subjective (7). Functional MRI, such as dynamic contrast-enhanced (DCE) MRI and diffusion-weighted imaging (DWI), is a promising method in assessing inflammatory changes at the involved sites in arthritis, given the potential for multiparametric information (8, 9). DCE-MRI is an imaging technique used to investigate the microvascular structure and function by recording a variety of physiological factors, such as tissue perfusion, arterial input function, capillary surface area, capillary permeability, and the volume of the extracellular extravascular space (EES) through tissue perfusion and permeability (*K*^trans^), extravascular extracellular volume fraction (*V*_e_), blood plasma volume (*V*_p_), and the efflux rate constant (*K*_ep_ = *K*^trans^/*V*_e_) (10). However, DCE-MRI is not routinely used in SpA because of the increased risk of renal fibrosis, allergies to gadolinium-based contrast agents (GBCAs), and deposition of residual gadolinium in brain, liver, skin, and bone (11, 12).

In DWI single exponential model, the influence of capillary microcirculation on apparent diffusion coefficient (ADC) value increases as the *b* value decreases while the signal-to-noise ratio (SNR) of MRI imaging decreases as the *b* value increases. Therefore, the diagnostic effectiveness of DWI single exponential model is limited (13). Intravoxel incoherent motion (IVIM) DW-MRI can measure tissue microcapillary perfusion by respectively recording pure diffusion coefficient (*D*), incoherent perfusion related microcirculation (D^*^), and microvascular perfusion fraction (*f*) using multiple *b*-values according to the double exponential curve (14). IVIM-derived perfusion parameters were expected to correlate with the kinetic features from DCE-MRI. However, it has been controversial whether the perfusion parameters are related with IVIM DWI and DCE-MRI (15). In AS, Zhao et al. found a moderate correlation (16). However, the parameters from DCE were semiquantitative, and there is no clear explanation for the correlation between hemodynamic parameters and underlying physiology (17, 18).

The purpose of this study was to determine whether there is a correlation between quantitative parameters of DCE-MRI and IVIM DWI from patients with axSpA, and to explore the factors that influence the correlation.

## Materials and Methods

### Patients

**Figure 1:**
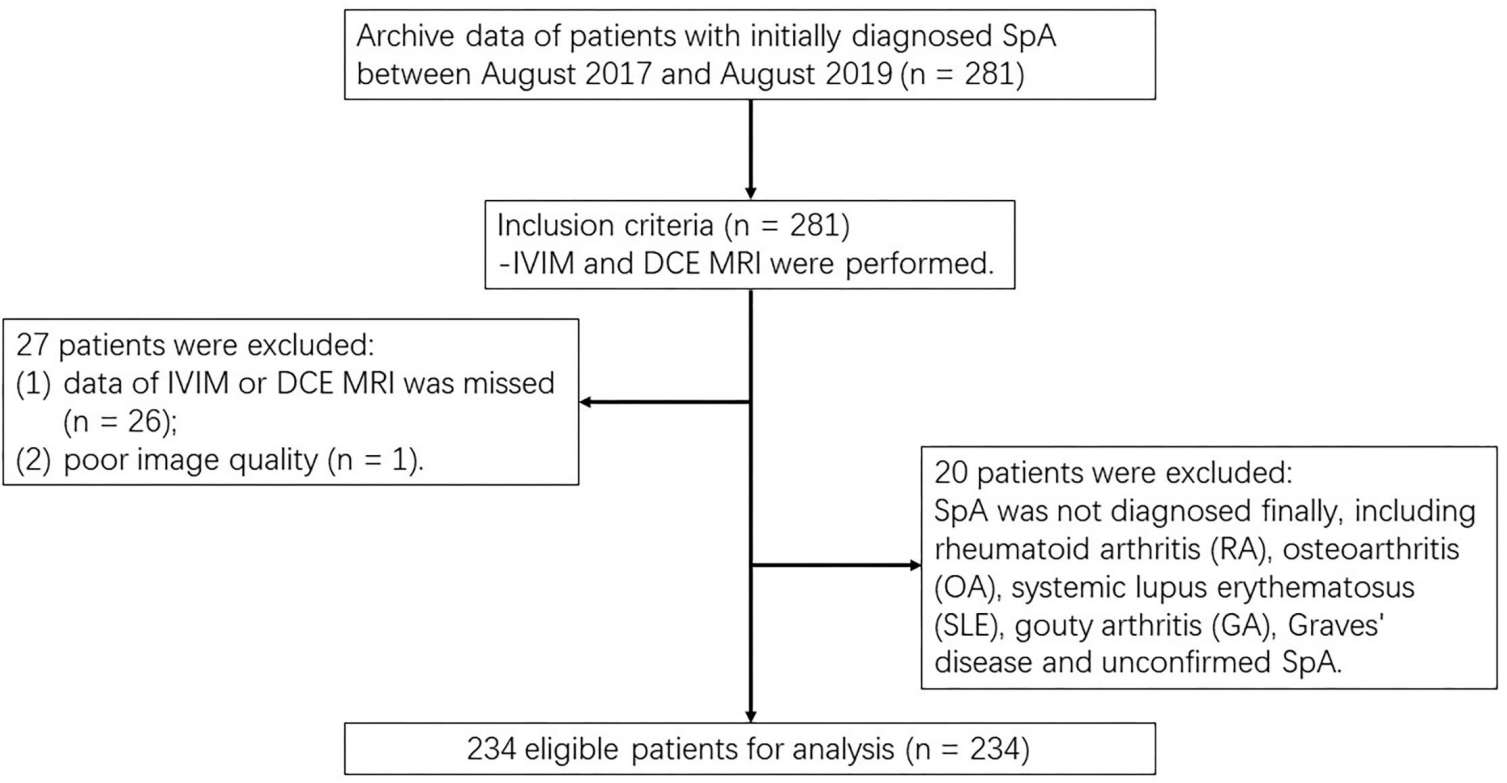
Flow diagram for patient selection.

This prospective study obtained approval from the institutional review board and each participant signed a written informed consent form (IRB number AN16327-001). Within a week after the laboratory tests and clinical assessments, MRI of the sacroiliac joints (SIJs), from August 2017 to August 2019. The inclusion criteria were as follows: (i) according to Assessment of Spondyloarthritis International Society classification criteria, axSpA was initially diagnosed (19); and (ii) both IVIM DWI and DCE-MRI were obtained. The exclusion criteria were defined as: (i) other diseases were identified, and the final diagnosis was confirmed as non-SpA, for example, rheumatoid arthritis (RA), osteoarthritis (OA), systemic lupus erythematosus (SLE), gouty arthritis (GA), and Graves' disease; (ii) image data were missing; and (iii) the SNR of MRI imaging was poor (Figure 1).

### MRI Techniques

MRI was performed using a clinical 3.0T system (Achieva 3.0T, Philips Healthcare, Best, Netherlands). The following four conventional sequences were implemented. (A) Axial T1-weighted turbo spin echo (T1-TSE), (B) axial T2-weighted spectral attenuated inversion recovery (T2W-SPAIR), (C) coronal T1-weighted mDixon turbo spin echo (T1W-mDixon-TSE), and (D) coronal T2-weighted mDixon turbo spin echo (T2W-mDixon-TSE). For IVIM DWI, a total of seven b values were used. Axial DCE-MRI was obtained using a three-dimensional T1 fast field echo (FFE) sequence with axial fat suppression. Two pre-contrast scans were performed before the contrast agent was injected, the third scan was performed at the same time as an intravenous injection of gadobutrolum (Gadovist, Bayer, Germany) with a dose of 0.1 mmol/kg and a flow rate of 2.5 ml/s. Each scan takes about 4.2 min. Scan parameters of IVIM DWI and DCE-MRI are summarized in Table 1.

**Table 1:** Intravoxel incoherent motion diffusion-weighted imaging (IVIM DWI) and DCE MRI parameters.

**Parameter**	**DCE MRI**	**IVIM DWI**
Orientation	Axial bilateral	Axial bilateral
Repetition time (msec)	3.5	3,594
Echo time (msec)	1.68	63
Flip angle (°)	8.15	90
Voxel size (mm^2^)	1.6 × 1.6	3 × 3
Fat suppression	Dixon	SPAIR
Field of view (mm^2^)	220 × 183	300 × 360
Matrix	140 × 114	100 × 120
Slice thickness (mm)	3	4
No. of signal acquired	12	6
Bandwidth (Hx/pixel)	541	2,668
Imaging time (min)	4.2	2.7
*b* values (s/mm^2^)	-	0, 10, 20, 30, 50, 600, 800

*DCE MRI, dynamic contrast-enhanced magnetic resonance imaging; IVIM DWI, Intravoxel incoherent motion diffusion-weighted imaging*.

### Image Analysis

Intravoxel incoherent motion diffusion-weighted imaging data were processed by the manufacturer-supplier (PRIDE DWI Tool, IDL Virtual Machine Version 6.3, Philips Healthcare, Japan) and Image J software (Version 1.46, Bethesda, MA, USA) to acquire the IVIM parameters apparent diffusion coefficient (*D, f*, and D^*^). Based on the IVIM DWI theory that Le Bihan et al. suggested, DWI with multiple *b*-values is performed in a double exponential model as Eq. 1 (20):


(1) $$\begin{array}{l} \frac{S_{b}}{S_{0}}=\;\left(1-f\right)\exp\left(-bD\right)+fexp\left[-b\left(D^{*}+D\right)\right] \end{array}$$

where *b* represents the diffusion gradient, *S*_*b*_ represents pixels whose signal intensity has diffusion gradient and *S*_0_ represents pixels whose signal intensity has no diffusion gradient (21). A simplified linear fitting equation with a value of *b* greater than 200 s/mm^2^ is used to obtain *D* as Eq. 2 (22):


(2) $$\begin{array}{l} S_{b}=S_{0}\exp\left(-bD\right) \end{array}$$

Where *f* and D^*^ were calculated using all *b* values by substituting *D* into Eq. 1 (23). The fitting performance is evaluated as:


(3) $$\begin{array}{l} R_{2}=1-SSE/SS_{total} \end{array}$$

where *SSE* represents the sum of squares errors between fitted curve and *SS*_*total*_ is defined as the sum of squares errors between all data and their overall mean.

Dynamic contrast-enhanced data were transmitted to a post-processing image workstation (Philips IntelliSpace Portal, Version 8.0, Philips Healthcare, Netherlands) for analysis. Pharmacokinetic analysis of the two-compartment model DCE-MRI applied to the linear version developed by Murase (24) can be obtained from the extended TK model (25). T1 mapping was calculated by measuring at two different flip angles, which were 8 and 15 degrees in our study.

### Patient Grouping for Assessment of the Factors Affecting the Correlation Between Quantitative Parameters From IVIM DWI and DCE-MRI

To thoroughly explore the factors which could affect the correlation between parameters from IVIM DWI and DCE-MRI, patients were divided into different groups based on six factors, such as demographic characteristics and features of the lesion progression. Patients were divided into the bone marrow edema (BME) group and non-BME group according to whether BME appears or not in their SIJs. Regardless of whether the lesion occurred on the left or right, patients with lesions on sacrum or ilium in SIJ were assigned to the sacrum or ilium group, and those with lesions on both sacrum and ilium were enrolled in the sacrum and ilium group. Based on the location of lesions, patients were classified into two groups: unilateral and bilateral groups. Depending on the course of this disease, two groups of patients were identified: <3 and ≥3 years groups. In addition, patients were divided into two groups according to age (<40-year-old vs. ≥ 40-year-old) and gender (female vs. male), respectively.

### Image Post-processing and Region of Interest (ROI)

**Figure 2:**
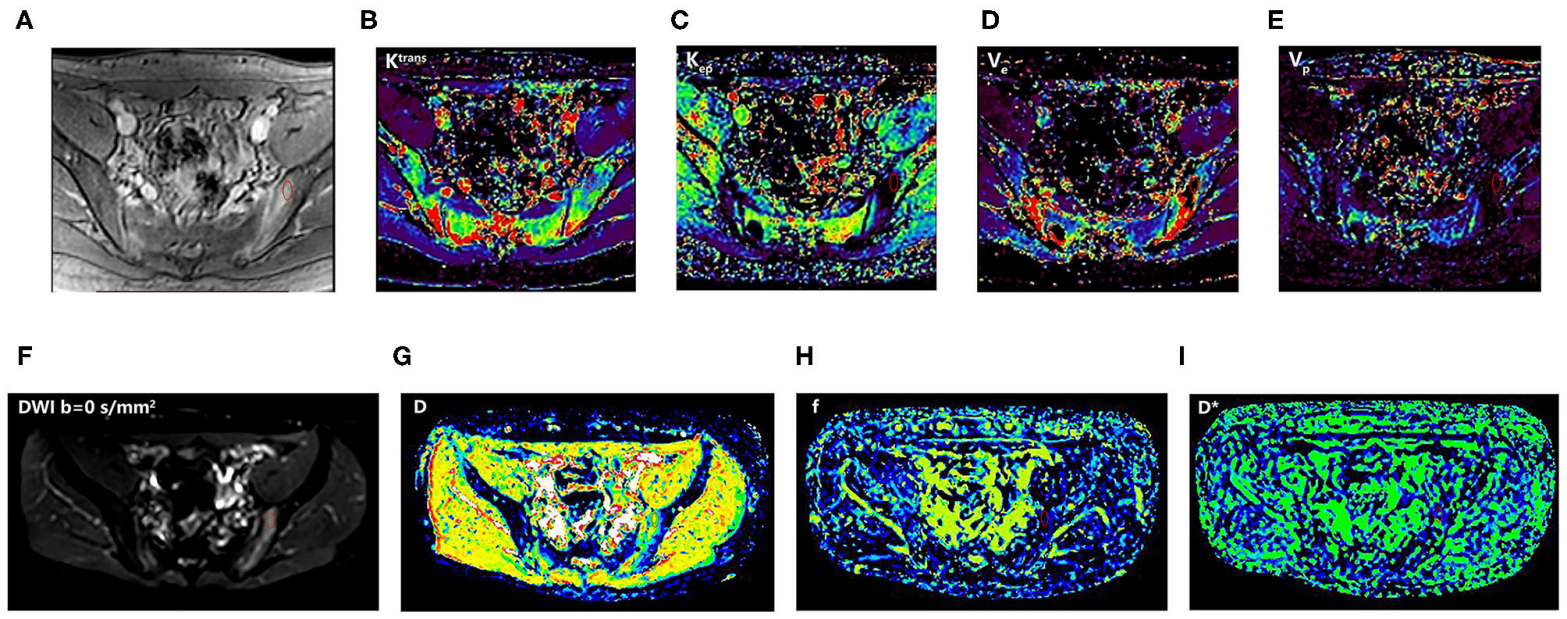
An 18-year-old man with SpA. The first-row images **(A–E)**: DCE-MRI and corresponding quantitative parameter images (*K*^trans^, *K*_ep_, *V*_e_, and *V*_p_) showing the position of ROI; the second-row images **(F–I)**: corresponding DWI (*b* = 0 s/mm^2^) and IVIM-DWI parameter images (*D, f*, and D*). SpA, spondyloarthritis; ROI, region of interest; T2W_SPAIR, T2-weighted spectral attenuated inversion recovery; DWI, Diffusion-weighted imaging; IVIM, intravoxel incoherent motion; *D*, pure diffusion coefficient; *f*, perfusion fraction; D*, pseudo-perfusion coefficient; DCE-MRI, dynamic contrast-enhanced magnetic resonance imaging; *K*^trans^, endothelial transfer constant; *K*_ep_, reflux rate; *V*_e_, fractional extravascular extracellular space volume; and *V*_p_, fractional plasma volume.

The DCE-MRI data were analyzed based on different region of interests (ROIs) by using the arterial input function (AIF) founded by Benjaminsen et al. (26). To verify the reliability of the parameter values, the three sets of ROIs were independently drawn on DCE images from 50 patients who were randomly selected by two radiologists with 10 and 3 years of experience in the musculoskeletal image interpretation, respectively (Radiologist 1 had 2 sets). They were blinded to each other measurements and the clinical and pathological characteristics of patients. Based on the size and position of ROIs on DCE images, the same ROIs were manually matched on DW images with *b*-values of 0 during Image J software processing (Figure 2). Details of obtaining ROIs of all patients were as follows.

For BME, ROIs were manually drawn inside the most obvious lesion at its maximum transverse level. For non-BME, ROIs were positioned in the area at the bone marrow under the articular surface of ilium or sacrum. To reduce the impact of the method of ROI selection on the conclusions, only one ROI is performed for evaluation with minimum contaminations from unintended tissues, such as blood vessels, bone cortex, cystic areas, necrosis, and fat deposition areas.

### Statistical Analysis

Statistical analysis of all data was performed by the Statistical Product and Service Solutions (SPSS) version 23.0 (IBM, Armonk, NY). To analyze normality and homogeneity of variance, the Shapiro–Wilk test and the Levene test were performed, respectively. Parameters from IVIM and DCE-MRI were reported as the mean ± SD. Intraclass correlation coefficient (ICC) and the coefficient of variation (CV) were calculated to evaluate intra- and inter-observer reliability using one-way (ANOVA).

A univariable linear regression analysis was performed to determine the correlation between IVIM MRI parameters and potential confounders, and DCE-MRI parameters. The value of *p* < 0.1 indicated that the result is statistically significant. Multi-variable linear regression analysis was used to assess the correlation between parameters from IVIM and those from DCE-MRI after controlling for the potential confounders. The value of *p* < 0.05 was considered as a marker of a statistically significant difference. Results are reported using regression coefficients and 95% *CI*s (α level, 0.05). Pearson's correlation coefficient (*r*) was calculated to measure the correlations above. A Bonferroni correction was conducted to protect from Type I error. The value of *p* < 0.0167 was considered statistically significant.

## Results

### Patient Characteristics

Our study initially included 281 patients with the initial diagnosis of axSpA, 26 patients were excluded due to data deficiency, 20 were excluded due to final diagnosis of non-SpA, and 1 patient was excluded due to poor image quality. Finally, 234 patients were enrolled (178 men, 56 women; mean age, 28.51 ± 9.50 years, and range, 12–64 years). The Shapiro–Wilk test showed that all parameters (*D, f*, D^*^, *K*^trans^, *K*_ep_, *V*_e_, and *V*_p_) were normally distributed (all *p* > 0.05), and the Levene's test revealed that all parameters were homogeneous (all *p* > 0.05). Table 2 summarized the mean and SD values of the IVIM and DCE parameters for each of the clinical-pathologic features of 234 patients with axSpA.

**Table 2:** Intravoxel incoherent motion (IVIM)-derived parameters according to clinical-pathologic features of 234 spondyloarthritis (SpA).

**Parameter**		**Men**	**Women**	***P* Value**
*D* (×10^−3^ mm^2^/s)		0.5 ± 0.4	0.5 ± 0.4	0.296
*f* (%)		10.2 ± 6.4	8.9 ± 5.5	0.192
D^*^ (×10^−3^ mm^2^/s)		127.8 ± 56.8	104.5 ± 59.2	0.009
*K*^trans^ (/min)		2,091.2 ± 1,354.8	2,586.0 ± 2,297.0	0.134
*K*_ep_ (/min)		1284.7 ± 912.8	1,235.8 ± 820.6	0.722
*V*_e_		5,136.3 ± 15,065.5	3,974.8 ± 5,861.1	0.575
*V*_p_		545.2 ± 634.9	641.6 ± 790.6	0.354
Age (y)^*^	<40 years	162 (91%)	43 (77%)	-
	≥40years	16 (9%)	13 (23%)	
Disease duration (m)^*^	≤ 3 years	90 (51%)	34 (61%)	-
	>3 years	88 (49%)	22 (39%)	
Lesion^*^	BME	37 (21%)	15 (27%)	-
	Non-BME	141 (79%)	41 (73%)	
Lesions in articular surfaces^*^	Sacrum or ilium	17 (10%)	9 (16%)	-
	Sacrum and ilium	161 (90%)	47 (84%)	
Scope of lesions^*^	Unilateral	11 (6%)	2 (4%)	-
	Bilateral	167 (94%)	54 (96%)	

Except where indicated, data are means ± SDs. D, pure diffusion coefficient; f, perfusion fraction; D^*^, pseudo-perfusion coefficient; K^trans^, endothelial transfer constant; K_ep_, reflux rate; V_e_, fractional extravascular extracellular space volume; V_p_, fractional plasma volume; BME, bone marrow edema.

* *Data are numbers of participants, with percentages in parentheses*.

### Intra- and Inter-observer Reproducibility

Table 3 indicated that interobserver reproducibility for IVIM perfusion-related parameters and DCE-MRI quantitative parameters ranges from good to excellent (ICC = 0.864–0.955, 0.981–0.994, respectively).

**Table 3:** Intra- and interobserver reproducibility in the assessment of IVIM and DCE-MRI parameters in sacroiliitis with ankylosing spondylitis (AS).

**Parameters**	**Intra- and interclass** **coefficient correlation** **(95%CI)**		**Coefficient of variation (%)**	
	**Intraobserver**	**Interobserver**	**Intraobserver**	**Interobserver**
*K*^trans^ (/min)	0.978 (0.963–0.988)	0.982 (0.969–0.990)	9.86	12.83
*K*_ep_ (/min)	0.990 (0.982–0.994)	0.994 (0.990–0.997)	20.30	20.76
*V*_e_	0.987 (0.977–0.993)	0.981 (0.967–0.989)	30.17	53.50
*V*_p_	0.956 (0.924–0.975)	0.981 (0.967–0.989)	55.79	54.40
*D* (×10^−3^ mm^2^/s)	0.959 (0.924–0.977)	0.955 (0.911–0.976)	4.03	6.20
*f* (%)	0.902 (0.833–0.943)	0.940 (0.879–0.968)	40.34	36.32
D^*^ (×10^−3^ mm^2^/s)	0.834 (0.725–0.902)	0.864 (0.761–0.923)	15.35	15.68

K^trans^, endothelial transfer constant; K_ep_, reflux rate; V_e_, fractional extravascular extracellular space volume; V_p_, fractional plasma volume; D, pure diffusion coefficient; f, perfusion fraction; D^*^, pseudo-perfusion coefficient; CI, confidence interval.

### Correlation Analyses of Parameters Derived From IVIM DWI and DCE-MRI

Results of the univariable regression analysis were shown in Table 4. Our study revealed that *D* was correlated with *K*^trans^, *K*_ep_, and *V*_e_ (all *p* < 0.1). For perfusion parameters, D^*^ was positively associated with *K*^trans^, whereas *f* was negatively associated with *K*^trans^ (both *p* < 0.1). Whether BME appears or not was a potential confounder as it was also correlated with *K*^trans^, *K*_ep_, and *V*_e_ (all *p* < 0.1). Gender and disease duration had correlations with *K*^trans^ and *K*_ep_ (*p* = 0.050 and 0.060, respectively). In addition, scopes of lesions were considered as potential confounders owing to their correlations with *K*^trans^ and *K*_ep_ (*p* = 0.020 and 0.033, respectively). However, there were no significant correlation between *V*_p_ and parameters from IVIM DWI.

**Table 4:** Results of univariable lines regression analyses.

**Parameters**	***K*^trans^ (/min)**	***K*_ep_ (/min)**	***V*_e_**	***V*_p_**
*D* (×10^−3^ mm^2^/s)	27.967 (23.871, 32.064) [<0.001^*^]	−9.349 (−12.056, −6.643) [<0.001^*^]	136.800 (95.712, 177.887) [<0.001^*^]	−0.558 (−2.806, 1.690) [0.625]
*f* (%)	−35.102 (−69.018, −1.186) [0.043^*^]	12.185 (−6.308, 30.678) [0.196]	−82.655 (−362.534, 197.223) [0.561]	−0.182 (−14.266, 13.901) [0.980]
D^*^ (×10^−3^ mm^2^/s)	0.032 (−0.005, 0.068) [0.087^*^]	0.006 (−0.013, 0.026) [0.528]	0.099 (−0.200, 0.398) [0.515]	<0.001 (−0.015, 0.015) [0.979]
Lesions (BME or Non-BME)	2,296.640 (1,880.161, 2,713.118) [<0.001^*^]	−821.809 (−1,078.121, −565.496) [<0.001^*^]	11,395.616 (7,476.339, 15,314.894) [<0.001^*^]	−24.850 (−235.381, 185.681) [0.816]
Age (y)	−448.451 (−1,091.301, 194.399) [0.171]	15.548 (−334.550, 365.645) [0.930]	−638.153 (−5,920.704, 4,644.397) [0.812]	−35.177 (−300.799, 230.446) [0.794]
Gender	−494.781 (−989.129, −0.433) [0.050^*^]	48.897 (−221.405, 319.199) [0.722]	1,161.471 (−2,915.841, 5,238.783) [0.575]	−96.442 (−301.226, 108.342) [0.354]
Sacrum and/or ilium	742.625 (119.767, 1,365.483) [0.020^*^]	−367.954 (−706.462, −29.447) [0.033^*^]	3,201.202 (−1,940.605, 8,343.009) [0.221]	−139.625 (−398.384, 119.135) [0.289]
Unilateral/ Bilateral	229.374 (−605.615, 1,064.363) [0.589]	−53.212 (−506.350, 399.926) [0.817^*^]	1,904.544 (−4,929.831, 8,738.919) [0.583]	−305.468 (−647.075, 36.139) [0.079^*^]
Disease duration (m)	−2.158 (−6.434, 2.118) [0.321]	2.217 (−0.090, 4.523) [0.060^*^]	−24.362 (−59.294, 10.570) [0.171]	−0.634 (−2.396, 1.128) [0.479]

Data are regression coefficients. Numbers in parentheses are 95% CIs, numbers in brackets are values of p. K^trans^, endothelial transfer constant; K_ep_, reflux rate; V_e_, fractional extravascular extracellular space volume; V_p_, fractional plasma volume; D, pure diffusion coefficient; f, perfusion fraction; D^*^, pseudo-perfusion coefficient; BME, bone marrow edema.

* *Data are statistically significant*.

**Figure 3:**
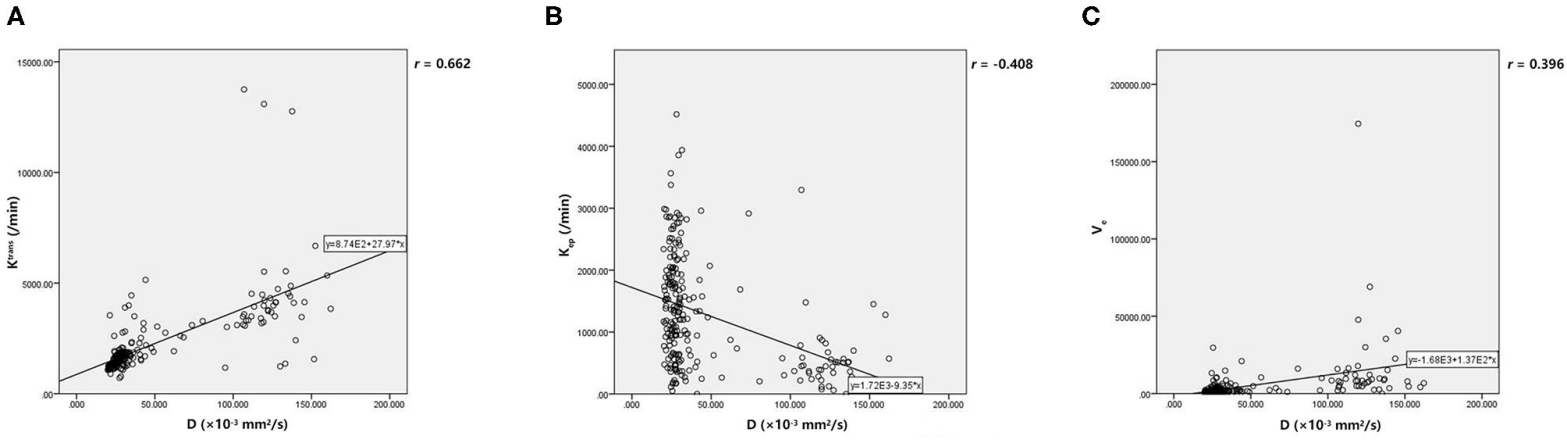
**(A, C)** The scatter plot showed that *D* have positive correlations with *K*^trans^ and *V*_e_. **(B)** There was negative correlation between *D* and *K*_ep_. *D*, pure diffusion coefficient; *K*^trans^, endothelial transfer constant; *K*_ep_, reflux rate; *V*_e_, fractional extravascular extracellular space volume.

Table 5 demonstrated that *D* was independently correlated with *K*^trans^, *K*_ep_, and *V*_e_ after adjusting for confounders using multivariable linear regression (all *p* < 0.05). However, *f* and D^*^ did not correlate with the parameters from DCE MRI after adjusting for confounders using multivariable linear regression (all *p* > 0.05). Finally, Pearson's correlation coefficient (r) demonstrated that *D* had a strong positive correlation with *K*^trans^ (*r* = 0.662, *p* < 0.001), a moderate negative correlation with *K*_ep_ (*r* = −0.408, *p* < 0.001) and a poor positive correlation with *V*_e_ (*r* = 0.396, *p* < 0.001). The results of Pearson's correlation analysis are shown in the scatter plots in Figure 3.

**Table 5:** Results of multivariable regression analyses.

**Parameters**	***K*^trans^ (/min)**	***K*_ep_ (/min)**	***V*_e_**
*D* (×10^−3^ mm^2^/s)	27.593 (18.968, 36.218) [<0.001^*^]	−6.707 (−12.409, −1.006) [0.021^*^]	131.074 (44.669, 217.480) [0.003^*^]
*f* (%)	−7.761 (−35.994, 20.472) [0.589]	-	-
D^*^ (×10^−3^ mm^2^/s)	0.021 (−0.010, 0.051) [0.183]	-	-

Data are regression coefficients. Numbers in parentheses are 95% CIs, numbers in brackets are values of p. Ktrans, endothelial transfer constant; K_ep_, reflux rate; V_e_, fractional extravascular extracellular space volume; V_p_, fractional plasma volume; D, pure diffusion coefficient; f, perfusion fraction; D^*^, pseudo-perfusion coefficient.

* *Data are statistically significant*.

## Discussion

Based on the large-scale samples (234 patients with axSpA), this study analyzed quantitative parameters that were superior to semi-quantitative parameters from DCE-MRI to assess the disease activity (7). We demonstrated correlations of quantitative parameters from IVIM DWI (*D, f*, and D^*^) and DCE-MRI (*K*^trans^, *K*_ep_, *V*_e_, and *V*_p_) in axSpA. We found that only *D* is correlated with the parameters from DCE-MRI. Our results revealed that their correlations were affected by BME, agenda, scopes of lesions, and course of the disease, except for age and localization of lesions.

In our study, the extended Tofts-Kety (ETK) model was used to assess *K*^trans^, *K*_ep_, *V*_e_, and *V*_p_, which is applied to weakly vascularized tissues with well-mixed interstitium and highly perfused tissues (27). Compared with other common models, the extended Tofts model can effectively evaluate *V*_p_, *K*^trans^, and *V*_e_ at low temporal resolution (28). Ye et al. (29) indicated that IVIM parameters generated by least-squares (LSQ) and Bayesian shrinkage prior (BSP) close to the actual values when using enough number of low *b*-values (0 < *b* < 50 s/mm^2^). There is a liver IVIM study showed that D^*^ values would be underestimated when the number of *b*-values is too small (30). In addition, the article suggested that liver IVIM studies should include at least two low *b*-values. However, in clinical practice, the scanning time should not be too long considering the physical conditions of patients and the need of diagnosis. Five low and two high b values in this study can reduce the scan time and meet the requirements of IVIM measurements. As a prospective study, the consistency of our data is relatively higher and the result has better clinical application value.

Our study found a strong positive correlation between *D* and *K*^trans^, and a poor positive correlation between *D* and *V*_e_. The results were consistent with our previous study that *D* had positive correlations with a few semi-quantitative DCE MRI perfusion-related parameters (11, 16). Quantitative DCE-MRI can not only measure the flow of local blood but also reflect the actual perfusion at the microscopic level (10). *K*^trans^ and *K*_ep_ reflect a complex combination of variables, such as blood flow, microvessel density (MVD), vascular endothelial growth factor (VEGF), and vascular permeability on the transport of contrast agent between the internal and external vessel (31). *D* detects the transportation of water molecules from capillary to marrow cavity in sacroiliitis in patients with axSpA to indirectly detect the inflammatory cell infiltration and fluid accumulation (32, 33). Many studies have shown that the peripheral articular synovium involved in patients with SpA shows a more significant increase in angiogenesis compared with those in patients with RA (34–36). Wang et al. found that a significant pathological feature of early sacroiliitis is the formation of subchondral fibrovascular tissue. The most common of these pathological features was the formation of pannus, accompanied by infiltration of inflammatory cells (37). Therefore, the *K*^trans^, *K*_ep_, and *D* increased in areas of BME in the SIJs of patients with axSpA. However, there was a moderate negative correlation between D and *K*_ep_ in our study. This is probably because of the short scan time for DCE-MRI. A long scan time (≥10 min) required for DCE-MRI is necessary for an accurate estimation of *V*_e_ and *K*_ep_, according to the literature (38), considering the time it takes for the contrast agent to reach the equilibrium between the EES and vascular space. *V*_e_ reflects the fractional extravascular extracellular space volume, which is affected by some factors, such as necrosis, cytogenesis, and leakage of contrast medium (39). The active stage of sacroiliitis is characterized by the destruction of cartilage and bone, which might increase *V*_e_ values.

Results of the univariable regression analysis revealed that perfusion parameters derived from IVIM DWI (*f* and D^*^) were associated with *K*^trans^ (both *p* < 0.1). However, there was no significant correlation between them after controlling confounders, such as BME, gender, and scopes of lesions. These results indicate that the correlations between perfusion parameters and *K*^trans^ were more affected by the confounders. D^*^ values reflect the mean capillary segment length and endovascular blood flow velocity, while *f* values reflect the volume of vascular and extracellular space (40, 41). Bray et al. found no significant difference in f and D^*^ between normal and inflamed marrow. In addition, their research showed that the enhancement with contrast in areas of BME may be due to an increase in capillary permeability instead of perfusion (42). Parameters derived from DCE-MRI ultimately reflect microvascular structure and function, which means that the perfusion mechanism indicated by K^trans^ and perfusion parameters derived from IVIM DWI is not exactly the same. Therefore, we could observe the increase of *K*^trans^ in areas of BME, while the change of perfusion parameters (*f* and D^*^) was not consistent with it. When evaluating hepatic injury induced by intestinal ischemia–reperfusion, Yang et al. found that *f* gradually decreased with the progress of reperfusion, while *K*^trans^ gradually increased, indicating that the presence of microcirculatory disorder and increased vascular permeability caused by inflammatory cytokines in the liver. Our study found that *f* was negatively correlated with *K*^trans^, which perhaps because of increased capillary permeability in the area of BME and microcirculatory disorder due to cell edema and inflammatory cell aggregation (43). Some studies have shown that there are differences in aspects of immunology, genetics, and hormone between men and women, such as higher circulating IL-17A and tumor necrosis factor-alpha (TNF-α) levels in men than in women with AS, which may affect the inflammatory pathways leading to bone damage and ultimately influence the progression of the radiographic changes (44, 45). As mentioned above, *K*^trans^ values obtained in this study were different in gender groups. The effect of the scopes of lesions shown in the results on perfusion is relevant to the special physiological anatomy of the SIJ. Compared with the sacrum side, the pannus invasion was more easily done on ilium side so that the vascularization, inflammatory cell infiltration, and formation of BME under the surface of the bone appeared earlier, resulting in a more serious inflammation (46).

Intra- and inter-observer reproducibility of parameters showed a large CV for *f* values, which represented the limited reproducibility of *f*. This is consistent with the results confirmed by Klaassen et al. (47).

Studies have shown that AIF has a large effect on calculating pharmacokinetic parameters accurately (27, 48, 49). Theoretically, the observer should select a local AIF sampled at the inlet to the target tissue. In this study, we selected the branch of the internal iliac artery adjacent to the areas of BME to define AIF. However, the slice orientation, flow artifacts, partial volume effect, and uncertain flow direction of the internal iliac artery affected the definition of AIF.

The major strength of this study was the large sample size of 234 patients, which reflected more reliable results compared with previous quantitative imaging studies of axSpA. However, this study still has some limitations. First, during the post-processing of DCE-MRI and DWI images, the ROIs of different sequences were matched manually by the observer, and the measurement of parameters was completed by different software, so there were different degrees of error between the ROIs of the extracted parameters. Second, the optimal *b* value in the IVIM examination has not been determined, so the optimization of *b* values needs to be further explored in future studies. Third, the time of DCE-MRI scanning protocol was short due to the limitation of clinical examination in this study, which may affect the accuracy of *V*_e_ and *K*_ep_. Fourth, in cases with a long course of the disease, there are different degrees of fat deposition in the affected areas, which will also affect the values of *V*_e_ and *V*_p_. In addition, our study did not group patients for disease activity, as some of the patients who received medication before MRI went into remission but still had BME in the sacroiliac joints on MRI.

The importance of structural lesions for diagnosis and follow-up is increasing, such as erosion, sclerosis, fat infiltration, and backfill (5). This study focused on the parameters of the areas of BME, but did not consider the presence of invisible microscopic structural lesions in the ROIs.

In conclusion, this study found that *D* derived from IVIM DWI was independently correlated with perfusion parameters derived from DCE-MRI, such as *K*^trans^, *K*_ep_, and *V*_e_. The correlations between perfusion parameters derived from IVIM DWI and DCE-MRI were influenced by BME, gender, and scopes of lesions.

## Data Availability Statement

The raw data supporting the conclusions of this article will be made available by the authors, without undue reservation.

## Ethics Statement

The studies involving human participants were reviewed and approved by Institutional Review Board of the Third Affiliated Hospital of Southern Medical University. Written informed consent to participate in this study was provided by the participants' legal guardian/next of kin.

## Author Contributions

CG and KZ contributed to the conceptualization, methodology, validation, investigation, data curation, and writing—original draft. QY contributed to the software and data curation. ZL contributed to methodology and writing—review and editing. ZX contributed to the investigation. XL contributed to the data curation and investigation. YZ contributed to the conceptualization, methodology, formal analysis, visualization, validation, and writing—review and editing. All authors contributed to the article and approved the submitted version.

## Funding

This study was supported by the National Natural Science Foundation of China (Grant Nos. 81871510, 82172014).

## Conflict of Interest

The authors declare that the research was conducted in the absence of any commercial or financial relationships that could be construed as a potential conflict of interest.

## Publisher's Note

All claims expressed in this article are solely those of the authors and do not necessarily represent those of their affiliated organizations, or those of the publisher, the editors and the reviewers. Any product that may be evaluated in this article, or claim that may be made by its manufacturer, is not guaranteed or endorsed by the publisher.
